# Heat preparedness and exertional heat illness in Paralympic athletes: A Tokyo 2020 survey

**DOI:** 10.1080/23328940.2022.2147364

**Published:** 2022-11-17

**Authors:** Puck Alkemade, Hein A. M. Daanen, Thomas W. J. Janssen, Elizabeth Broad, Victoria L. Goosey-Tolfrey, Tatsuru Ibusuki, Hiske Kneepkens, Julien D. Périard, Thijs M. H. Eijsvogels

**Affiliations:** a Behavioural and Movement Sciences, Vrije Universiteit Amsterdam, Amsterdam Movement Sciences, Amsterdam, The Netherlands; b Sports Dietitian, Huskisson, New south wales, Australia; c Centre for Disability Sport, School of Sport, Exercise and Health Sciences, Loughborough University, Loughborough, Leicestershire LE11 3TU, UK; d Department of Rehabilitation Medicine, Akeno Central Hospital, Oita, Japan; e Sport Medisch Centrum Papendal, NOC*NSF, Arnhem, The Netherlands; f University of Canberra Research Institute for Sport and Exercise, Bruce, New south wales, Australia; g Radboud Institute for Health Sciences, Department of Physiology, Radboud University Medical Center, Nijmegen, The Netherlands

**Keywords:** Heat acclimation, cooling, heat mitigation, para-athletes, adapted sports, impairment

## Abstract

Paralympic athletes may be at increased risk for exertional heat illness (EHI) due to reduced thermoregulatory ability as a consequence of their impairment. This study investigated the occurrence of heat-stress related symptoms and EHI, and the use of heat mitigation strategies in Paralympic athletes, both in relation to the Tokyo 2020 Paralympic Games and previous events. Paralympic athletes competing in Tokyo 2020 were invited to complete an online survey five weeks prior to the Paralympics and up to eight weeks after the Games. 107 athletes (30 [24–38] years, 52% female, 20 nationalities, 21 sports) completed the survey. 57% of respondents had previously experienced heat-stress related symptoms, while 9% had been medically diagnosed with EHI. In Tokyo, 21% experienced at least one heat-stress related symptom, while none reported an EHI. The most common symptom and EHI were, respectively, dizziness and dehydration. In preparation for Tokyo, 58% of respondents used a heat acclimation strategy, most commonly heat acclimatization, which was more than in preparation for previous events (45%; *P* = 0.007). Cooling strategies were used by 77% of athletes in Tokyo, compared to 66% during past events (*P* = 0.18). Cold towels and packs were used most commonly. Respondents reported no medically-diagnosed EHIs during the Tokyo 2020 Paralympic Games, despite the hot and humid conditions in the first seven days of competition. Heat acclimation and cooling strategies were used by the majority of athletes, with heat acclimation being adopted more often than for previous competitions.

## Introduction

In the lead up to the Tokyo 2020 Olympic and Paralympic Games, many concerns were raised regarding the anticipated environmental conditions [e.g. [1–6]]. The Games, held in the Tokyo summer of 2021, were expected to be the most thermally challenging ever, with an air temperature of 31°C, relative humidity of 60% and wet-bulb globe temperature (WBGT) of 29°C during the hottest part of the days [1]. Hot and/or humid environments have a negative impact on exercise performance, and increase the risk for heat-stress related symptoms or exertional heat illness (EHI) [2–4]. Such environmental conditions are challenging for all athletes, but Paralympic athletes may be affected more than Olympic athletes [2].

It is well-established that thermoregulation is impaired in athletes with a spinal cord injury (SCI) when sudomotor and/or vasomotor pathways in the spine are damaged [2,5]. Wheelchair rugby athletes with a cervical SCI experience hyperthermia even in temperate environments, with an average core temperature increase of 1.6°C within four 8-min quarters of match play [6]. Few experimental data are available on thermoregulation in para-athletes with impairments other than SCI, but it has been suggested that they also experience impairment-related disadvantages when competing in the heat [2,7]. Individuals with amputations may show gait asymmetries and a reduced body-surface-area to mass ratio, resulting in elevated metabolic heat production and impaired ability to dissipate heat, respectively [2,8]. In addition, amputees may experience increased discomfort at the prosthetic socket barrier and/or skin grafts when exercising in the heat [2,9]. Athletes with visual or intellectual impairments are not expected to show an impaired thermoregulatory response, but reduced pacing and hydration awareness might lead to indirect disadvantages during competition in the heat [2,10].

There is a paucity of research on EHI incidence in elite para-athletes. During the 2015 World Para Athletics Championships in Doha (Qatar), where ambient temperature ranged between 25 and 36°C, seven of the 1225 athletes (0.6%) were diagnosed with EHI, with four of them being referred to the hospital [11]. It remains to be determined whether EHI risk is similar in other Paralympic sports. Of note, the Doha study did not report whether athletes used heat mitigation strategies [11]. The effectiveness of heat acclimation (HA) and cooling strategies in reducing thermal strain has been shown in para-athletes, predominantly in those with an SCI [12–15]. However, little is known about the use of such strategies in practice. Hence, additional research is warranted regarding EHI incidence in Paralympic athletes, as well as the use of heat mitigation strategies to lower EHI risk.

The aim of this study was to investigate the occurrence of self-reported heat-stress related symptoms and EHI, and the use of heat mitigation strategies in Paralympic athletes, both in relation to the Tokyo 2020 Paralympic Games and previous events. In addition, we explored the influence of participant characteristics on heat-stress related symptom occurrence and heat mitigation strategy use.

## Materials and methods

### Study design

The Paralympic survey was administered online (Qualtrics, Provo, Utah, USA) and was available in 10 languages (Arabic, Chinese, Dutch, English, French, German, Japanese, Portuguese, Russian, Spanish). Only athletes competing at the Tokyo 2020 Paralympic Games were eligible to participate in the study. The survey was brought to the attention of these athletes via advertisements on social media and the networks of the international group of researchers. Athletes were given the opportunity to complete a pre-Paralympic survey only, or a full Paralympic survey. The full Paralympic survey was either completed as a combination of the pre-Paralympic survey and a short post-Paralympic survey (Supplemental file 1), or completed in full after the Games. Surveys were open for participation five weeks prior to and eight weeks after the Paralympics. Before commencing the survey, respondents were informed about the purpose of the study and provided informed consent. Procedures were approved by the Ethics Committee of the Faculty of Behavioral and Movement Sciences of the Vrije Universiteit Amsterdam (#VCWE-2021-145) and conformed to the standards set out by the Declaration of Helsinki. After the Paralympic Games, meteorological data were derived from the Japan Meteorological Agency [16].

### Paralympic survey

The Paralympic survey included questions about impairment, sports participation, heat-stress related symptoms, EHI, HA, cooling and hydration strategies. Impairments were categorized in four groups: SCI, Limb deficiency, Visual impairment/intellectual impairment/Pilots, or Other (Supplemental file 2, Table 1). Athletes with visual or intellectual impairments were grouped with the pilots (n = 3), as they are physiologically similar. Sports disciplines were classified by environment (i.e. indoor or outdoor) and further categorized in one of four categories: Skill, Power, Mixed or Endurance [17] (Supplemental file 2, Table 2 and 3). Athletes competing in multiple disciplines were categorized based on the class with the highest expected thermoregulatory demands. Symptoms and strategies were asked in relation to the Tokyo 2020 Paralympic Games and previous events (also referred to as “in the past”). Most questions were multiple choice, though respondents were given the opportunity to expand on their response. Heat-stress related symptoms were listed as collapsing/fainting, cramping, dizziness, nausea, severe headache and vomiting. Medically-diagnosed EHIs were listed as dehydration, hyponatremia, heat syncope, heat exhaustion and heat stroke [18,19]. In the survey, it was articulated that the EHI diagnosis should have been made by first-aid personnel, a team physician, general practitioner, or in a hospital. In survey questions on cooling strategies, we differentiated between cooling prior to competition (*pre*-cooling) and cooling during competition (exercise or breaks; *per-*cooling). Cooling strategies were grouped into seven main categories (Supplemental file 2, Table 4). As we administered surveys both before (pre-survey) and after (post-survey and full survey) the Paralympics, preparatory strategies for the Tokyo 2020 Paralympic Games were either planned or actually used. We did not differentiate between planned or used strategies.

### Meteorological data

Meteorological data were collected from Tokyo (i.e. the city), where most Paralympic venues were located. Data included air temperature (°C), relative humidity (%), dew point temperature (°C), precipitation (mm), solar radiation (MJ·m^−2^) and wind speed (m·s^−1^). Hourly data was retrieved from August 18 (6 days prior to start Paralympic Games) until September 5 (last day of Paralympic Games) 2021. Daily parameters were calculated using a timespan 06:00–22:00 h, based on the Paralympic time schedule. WBGT was calculated from meteorological data according to the Liljegren method [20] using the R package “HeatStress” developed by MeteoSwiss [21].

### Data analysis

Data processing and analyses were performed using R software (version 4.1.1, R Foundation for Statistical Computing, Vienna, Austria) in the Rstudio environment (version 2021.09.0, Rstudio, Inc., Boston, MA, USA). The level of statistical significance was set at *P* < 0.05. Normality of data was tested using the Shapiro-Wilk test. Descriptive data were reported as mean ± standard deviation (in case of normal distribution) or median [first quartile–third quartile] (in case of non-normal distribution). The number of respondents (*n*) per question was stated throughout the results section. Frequencies from single-answer questions (only one answer allowed) were expressed in percentage of total respondents. Frequencies from multiple-answer questions (multiple answers allowed) were expressed in percentage of total answers selected. McNemar’s test was used to assess within-participant differences in the incidence of heat-stress related symptoms, HA use and cooling use reported in the past and in Tokyo. We performed simple ordinal and binary logistic regressions to investigate the influence of sex, age, continent, impairment category, competition environment and outdoor competition category on self-rated heat-coping ability, heat-stress related symptom occurrence, cooling and HA use. We also tested the influence of heat-stress related symptom history on strategy use in Tokyo, and the influence of strategy use on symptom occurrence in Tokyo. The odds ratio (OR) was reported with a 95% confidence interval. The category expected to have the lowest probability was chosen as reference category *a priori*.

## Results

### Respondents

**Figure 1.**
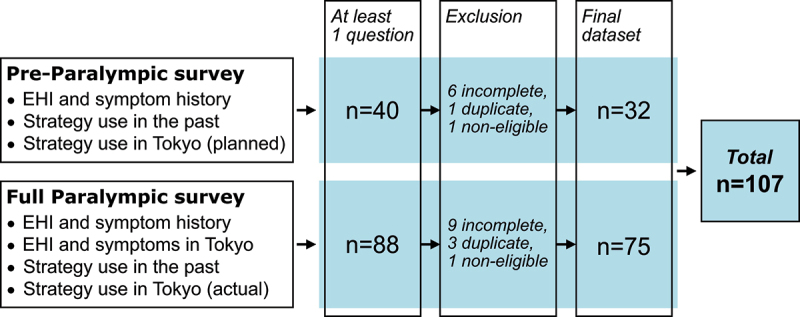
Overview of data collection and respondent selection. EHI, exertional heat illness.

**Figure 2.**
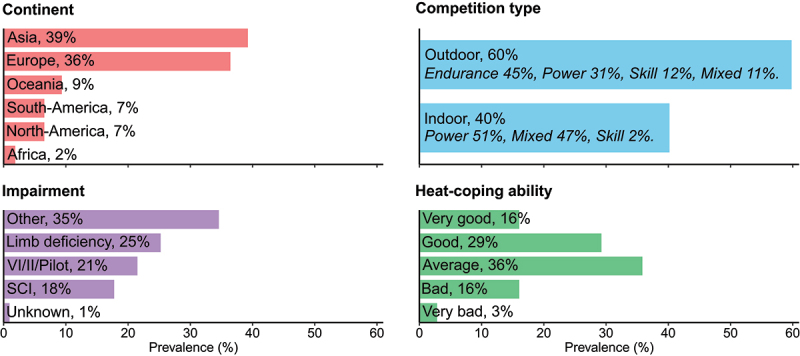
Respondent characteristics (n = 107). II, intellectual impairment; SCI, spinal cord injury; VI, visual impairment.

A total of 128 athletes participated in the online survey, but 21 were excluded due to limited answers, non-eligibility or duplicate responses (Figure 1). The final dataset included 107 respondents of 20 different nationalities (51 males, 56 females; Figure 2), with a self-reported age of 30 years [24–38 years], height of 171 cm [160–179 cm] and body mass of 64.9 ± 14.6 kg. Respondents reported 16 [9–18] training hours per week. Athletes from 21 different sports were included, with most common sports being athletics (*n* = 29; endurance *n* = 11, power *n* = 18), swimming (*n* = 16) and wheelchair basketball (*n* = 11).

### Meteorological data

**Figure 3.**
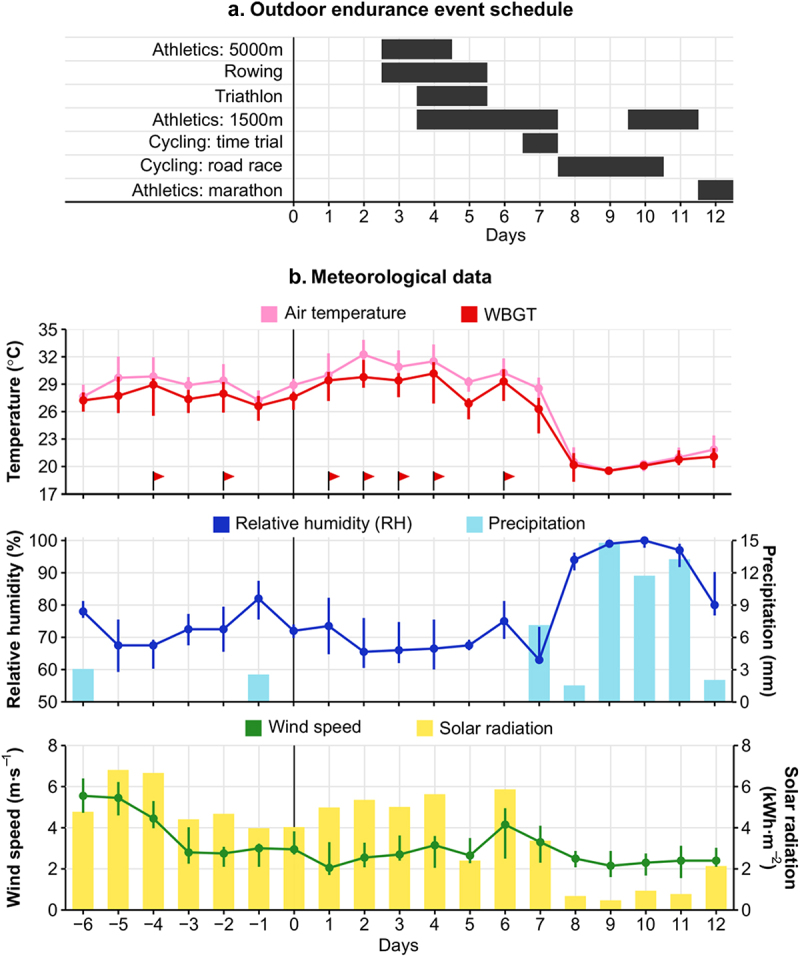
**a;** Schedule of the outdoor endurance events at the Tokyo 2020 Paralympic Games. **b**; Daily (06:00–22:00 h) meteorological data 6 days before and 12 days during the Tokyo 2020 Paralympic Games. Points and error bars represent median and interquartile range, respectively. Red flags highlight daily wet-bulb globe temperature (WBGT) medians that exceeded the “cancel level for exertional heat stroke risk” for competition as advised by The American College of Sports Medicine (>27.9°C) [43].

Meteorological variables during the Tokyo 2020 Paralympic Games are displayed in Figure 3. During the first seven days of competition in Tokyo, average daily WBGT, air temperature and relative humidity were respectively 28°C [27–30°C], 30°C [28.7–32.3°C] and 68% [63–77%]. At day 8, WBGT decreased, with during days 8 to 12 an average WBGT of 20°C [19–21°C], air temperature of 20.4°C [19.5–21.6°C] and relative humidity of 98% [91–99%].

### Heat-stress related symptoms and EHI

**Figure 4.**
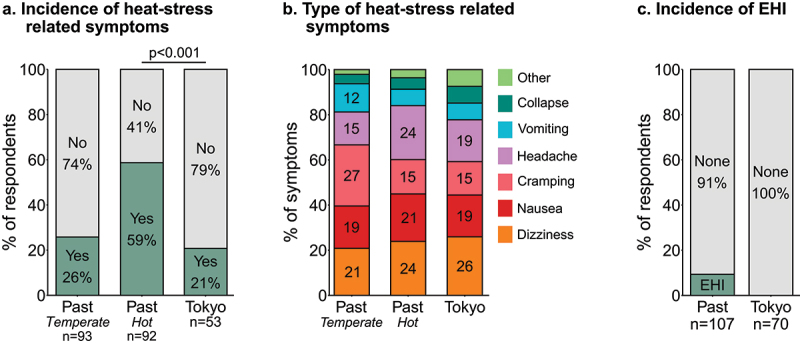
**a;** Incidence of heat-stress related symptoms during past events (temperate environment [15 to 25°C] and hot environment [>25°C]) and in Tokyo. **b;** Incidence of specific heat-stress related symptoms during past events and in Tokyo. **c;** Incidence of exertional heat illness (EHI) during past events and in Tokyo. Percentages >10% are displayed in the appropriate bars.

In both the past and in Tokyo, respondents experienced heat-stress related symptoms, with dizziness, nausea and severe headache being most prevalent in the heat (Figures 4a,b). Nine percent of respondents reported being medically diagnosed with EHI in the past at least once (Figure 4c), with dehydration (38% of EHIs reported) and heat exhaustion (31%) being most prevalent. None of the respondents reported being medically diagnosed with EHI in Tokyo.

### Heat acclimation

**Figure 5.**
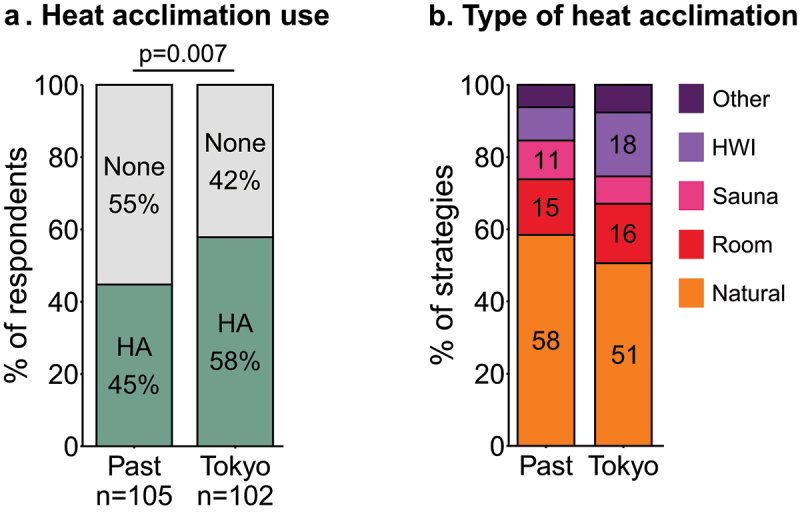
**a;** Prevalence of heat acclimation (HA) in preparation for past events and for Tokyo. **b;** Prevalence of specific HA strategies in preparation for past events and for Tokyo. Percentages >10% are displayed in the appropriate bars. HWI, hot water immersion; Room, artificial environment e.g. climate chamber or hot room.

In preparation for past events, 45% of respondents had used HA (Figure 5a), of which 70% had used one and 24% had used two different strategies. In preparation for Tokyo, HA was used by more respondents (58%) than in the past (*P* = 0.007). HA for Tokyo was most commonly performed in a natural environment (e.g. heat acclimatization during training camp), or using hot water immersion (Figure 5b). Most respondents employed one (68%) or two complementary HA strategies (26%) in Tokyo. HA strategies used to prepare for Tokyo included 12 [6.8–20] HA sessions distributed over 16 [6–22] days. In the year prior to the Tokyo 2020 Paralympic Games, respondents had resided in a hot climate (> 25°C) for less than two months (44%), four to six months (16%) or more than six months (25%), while 15% of respondents had not resided in a hot climate. Of note, some respondents may have naturally acclimatized without using a specific HA strategy; 43% of respondents who had resided in a hot climate for more than four months did not report to have used HA specifically. The most prevalent reason for not using HA in Tokyo was “I think/thought it was not needed for this event” (Table 1).

**Table 1.** Reasons for not using heat acclimation and cooling strategies in the past and in Tokyo, listed in order of decreasing prevalence.

	Heat acclimation		Cooling	
	Past (%)	Tokyo (%)	Past (%)	Tokyo (%)
*n respondents*	*57*	*42*	*34*	*19*
Not needed for this event	-	39	-	71
Never competed in warm/hot conditions	29	-	31	-
Insufficient knowledge	25	18	29	14
Did/does not fit in training schedule	17	11	-	-
Insufficient financial resources	13	15	13	0
Don’t like cooling	-	-	13	14
Other	10	11	4	0
Thought/think it was/is not effective	5	6	9	0

### Cooling

**Figure 6.**
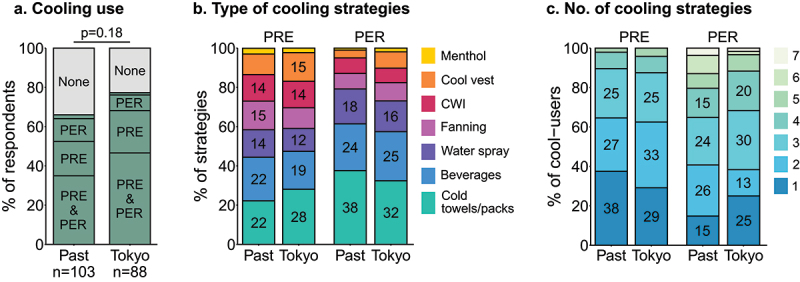
**a;** Prevalence of cooling strategy use during past events and in Tokyo. Cooling was used both pre- (PRE) and during (PER) competition, or only PRE or PER (small proportion not specified). **b;** Prevalence of specific PRE- and PER-cooling strategies during past events and in Tokyo. CWI, cold water immersion. **c;** Prevalence of number of different cooling strategies reported by cooling users during past events and in Tokyo. Percentages >10% are displayed in the appropriate bars.

In the past, 66% of respondents had used at least one cooling strategy (Figure 6a). In Tokyo, 77% of respondents used cooling, with most combining pre- and per-cooling. Both in the past and in Tokyo, cold towels/packs, beverages and water sprays were used most frequently (Figure 6b). In Tokyo, 87% of respondents used one to three pre-cooling strategies and 88% used one to four per-cooling strategies (Figure 6c). The most prevalent reason for not using a cooling strategy in Tokyo was “I think/thought it was not needed for this event” (Table 1).

### Hydration plan

In Tokyo, 71% of respondents had a hydration plan, either prior to or during competition (Supplemental file 3, Figure 1). The most prevalent fluids consumed were water (37% of all strategies reported), electrolyte drinks (30%) and fluids containing carbohydrates/sugars (16%).

### Strategy development Tokyo Paralympics

At the Tokyo 2020 Paralympic Games, 46% of respondents used a combination of HA, cooling and hydration strategies, while 29% used two, 17% one, and 8% none of these strategies. In developing strategies for Tokyo, respondents were primarily supported by a sport scientist, dietitian and/or physician (44%), coach (31%), and/or by themselves (16%; Supplemental file 3, Figure 2). The factors most influencing the use of strategies in Tokyo were previous experiences with the particular strategies (39%), the ability to integrate the strategy with regular training (17%) and recent research findings (14%; Supplemental file 3, Figure 2). Limitations due to COVID-19 regulations and financial resources were minimally influential (both 3%).

### Explanatory models

**Figure 7.**
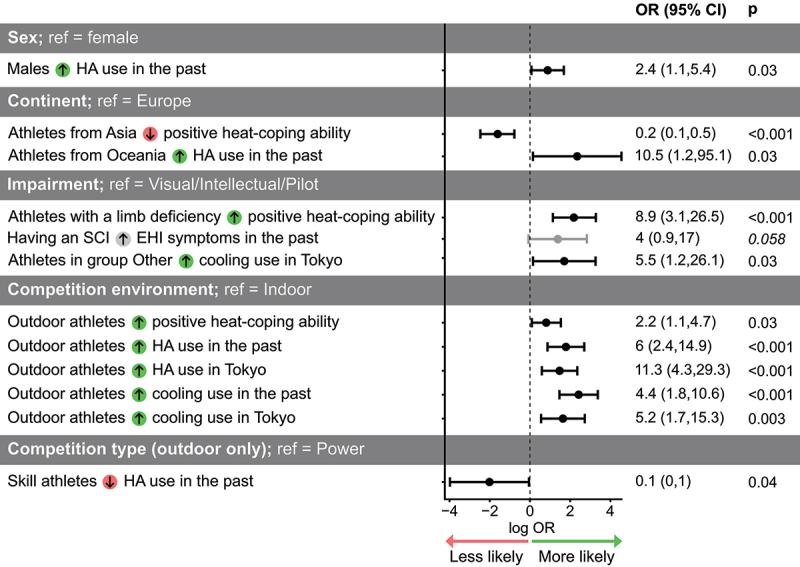
Simple logistic regression outcomes. The likelihood of the dependent variables was tested for the participant characteristics (predictor variables) shown in the table. Dependent variables were heat-coping ability rating, occurrence of heat-stress related symptoms, heat acclimation (HA) use, and cooling use in the past and in Tokyo. Green and red arrow symbols denote a higher and lower likelihood with respect to the reference variable (ref), respectively. Points and error bars represent odds ratio (OR) estimate and confidence interval (CI) on a logarithmic scale. Only outcomes with *P* < 0.06 are presented (significance level *P* < 0.05).

Athletes competing outdoors were more likely to use HA and cooling strategies than those competing indoors, both in the past and in Tokyo (Figure 7; Supplemental file 4). Respondents who experienced heat-stress related symptoms in the past were more likely to use HA and cooling strategies in Tokyo (OR = 2.4 (1, 5.9), *P* = 0.045; OR = 4.0 (1.3, 12.1), *P* = 0.01; respectively). HA or cooling strategy use in Tokyo did not influence heat-stress related symptom occurrence (*P* > 0.40).

## Discussion

The main objective of this study was to investigate the occurrence of heat-stress related symptoms and EHI, and the use of heat mitigation strategies in Paralympic athletes, both in relation to the Tokyo 2020 Paralympic Games and previous events. During previous events, 59% of athletes had experienced heat-stress related symptoms, while nine percent had been medically diagnosed with an EHI. In Tokyo, 21% of athletes experienced heat-stress related symptoms, while none reported a medically-diagnosed EHI. In preparation for Tokyo, HA was used by more athletes compared to previous events, with 58% using HA for Tokyo compared to 45% in the past. Cooling strategies were used more frequently than HA, with 66% of respondents indicating having used cooling strategies in the past and 77% in Tokyo. Respondents who experienced heat-stress related symptoms in the past were more likely to use heat mitigation strategies in Tokyo.

Notwithstanding the extreme environmental conditions in the first seven days of the Paralympics, none of the respondents reported being medically diagnosed with EHI. This finding is supported by media stating that during the Tokyo Paralympics only 15 out of the 4403 athletes (0.3%) reported to medical support with an heat-related illness [23,24]. The IPC Medical Committee confirmed to the authors that they registered 16 cases of hyperthermia or heat illness [Derman et al., under review]. The low EHI incidence in Tokyo aligns with observations at the 2015 World Para Athletics Championships in Doha, where 0.6% of athletes were diagnosed with a moderate to severe heat-related illness [11]. Furthermore, 21% of our respondents experienced at least one heat-stress related symptom during or immediately after competition, which is lower than reported in a previous study [7]. During a paratriathlon in the heat (33°C, 35–41% relative humidity), 16 out of 28 athletes (57%) reported heat-stress related symptoms, with half of the athletes having heat acclimated prior to competition [7]. However, paratriathlon is a high-intensity outdoor endurance sport, exposing athletes to an elevated risk for heat stress, while the athletes in the current study competed in multiple Paralympic sports with various environmental and physiological demands. A closer examination of our dataset indicated that three out of the 16 outdoor endurance athletes in the sample (19%) experienced heat-stress related symptoms during the Paralympics. Of note, the paracycling road races and the marathons were held in the last five days of the Games, during which the WBGT was considerably lower than the first seven days (see Figure 3). This likely lowered risk for EHI and heat-related symptoms for athletes competing in these sports [25].

The observation that none of the athletes in our study were diagnosed with EHI in Tokyo may also relate to the considerable attention afforded to the hot and humid conditions expected at the Tokyo 2020 Olympic and Paralympic Games. Several studies and reviews were published on expected weather conditions, EHI risk and management, and heat mitigation strategies [1,2,26–29]. Researchers and practitioners collaborated in national projects on heat mitigation (e.g. UK, “Project Theta”; Netherlands, “Citius Altius Sanius” and “Thermo Tokyo” [22,30]), and in developing translational documents to educate athletes about heat management [31,32]. The high prevalence of heat mitigation strategy use among athletes in Tokyo suggests that such initiatives successfully created awareness within athletes and their support teams. During the Paralympics, the organizing committee implemented various heat countermeasures, such as shading facilities, air-conditioned rooms, and fluid and ice supply [33–35]. Outdoor endurance competitions, such as the paratriathlon and marathon races, started at 6:30 in the morning to avoid the hottest part of the day [34]. Additionally, some international federations adopted WBGT limits for re-scheduling or canceling competition [31,36,37]. For example, multiple Paralympic tennis matches were suspended or play continued under a retracted roof in a climate-controlled setting (Center Court only) as the WBGT exceeded the cancellation limit (>30.1°C) [37].

Ninety-two percent of respondents used at least one heat mitigation strategy (i.e. HA, cooling or hydration strategy) in preparation for or during the Tokyo 2020 Paralympic Games. When interpreting HA use, one should bear in mind that some athletes resided in a warm climate, and therefore likely did not require or decide to use a HA strategy. Indeed, 41% of respondents spent more than four months in a hot climate in the year prior to Tokyo, with almost half not using a HA strategy. Limited research is available on the effectiveness of HA in Paralympic athletes. Both athletes with an SCI and para-triathletes with various impairments showed typical heat adaptations to a 7-8-day HA protocol, including lowered exercise aural or gastrointestinal temperature and expansion of plasma volume [12,14]. However, in both cases no sweat adaptation was observed, suggesting that para-athletes can successfully heat acclimate, but perhaps not to the extent observed in able-bodied athletes.

Two-thirds of respondents used cooling strategies to mitigate heat stress in Tokyo. Cold towels and packs were used most often, presumably due to their practical convenience. The efficacy of such strategies largely depends on the body surface area exposed to the cooling and the duration and temperature of cooling. The second most popular cooling strategy was ingestion of beverages such as cold water and ice slurry. It has been shown that ice slurry ingestion prior to exercise can reduce body core temperature in wheelchair rugby and basketball players with an SCI, but the cooling benefits may disappear during exercise [38,39]. Interestingly, cooling vests were less popular than cold towels and packs, beverages, and water sprays, even though wearing an ice vest may be one of the most effective strategies in athletes with an SCI [13,40]. The limited use of cooling vests may be due to their weight, and the uncomfortable fit for wheelchair athletes [13]. Most athletes used a combination of pre- and per-cooling, which is more effective than using only one of them [13,40–42]. Additionally, the majority of athletes used multiple pre- and per-cooling strategies, which has been shown more beneficial than single strategy use in able-bodied athletes [42]. Altogether, heat mitigation strategies have been and are commonly used by Paralympic athletes, despite the paucity of research focusing on this population. Additional studies are required to optimize the use of heat mitigation strategies in para-athletes, as findings in able-bodied athletes may not be applicable.

Our data suggests that sex, continent, impairment, competition environment or sports type did not influence heat-stress related symptom occurrence. In Paralympic athletes, risk for heat-stress related symptoms and EHI is associated with the environmental and physiological demands of the sport, but importantly, also to the impairment [2]. The combination of these factors may be more indicative of the risk for heat-stress related symptoms than each factor alone, but our sample size did not allow a more in-depth analysis. In our study, the risk for heat-stress related symptoms is further influenced by the inconsistent weather conditions during the Paralympics. Hence, our data is not suitable to create accurate risk profiles.

Our study is the first to provide insight into heat-stress related symptoms, EHI, and heat mitigation strategy use in Paralympic athletes across a wide range of sports. Although we included more than 100 respondents, this accounted for only 2.4% of all Paralympians [24]. Notwithstanding, we studied a diverse sample, with respondents from 20 different countries participating in 21 different sports. The survey was advertised through social media and the network of the researchers, which might have specifically targeted athletes who use modern technology and/or are supported by embedded scientists. These athletes may also be more likely to use heat mitigation strategies, so a potential effect of participation bias cannot be discarded. Lastly, for 40 respondents we only collected pre-Paralympic data, and therefore only obtained information on planned strategies rather than those that were actually used. Nonetheless, our data provide novel insights into heat mitigation strategy use in Paralympic athletes.

## Conclusions

During past events, more than half of the athletes participating in the study had experienced heat-stress related symptoms, while nine percent had been medically diagnosed with EHI. At the 2020 Tokyo Paralympic Games, despite the hot and humid conditions during the first seven days of competition, none of the respondents reported a medically-diagnosed EHI. Though, 21% experienced heat-stress related symptoms in Tokyo. HA and cooling strategies were widely used among Paralympic athletes. In preparation for Tokyo, HA was used by 58% of respondents, while only 45% had used HA during previous events. The abundant use of heat mitigation strategies and the countermeasures taken by the Tokyo 2020 organizational committee likely contributed to the absence of EHI during the Paralympics. Hence, although many Paralympic athletes may be at an increased risk of experiencing EHI due to their impairment, they can, with the appropriate strategies and countermeasures, safely compete in the heat.

## Supplementary Material

Supplemental Material

## List of abbreviations

EHI, exertional heat illness.

HA, heat acclimation.

SCI, spinal cord injury.

WBGT, wet-bulb globe temperature.
