# Supplementary material for: Heat preparedness and exertional heat illness in Paralympic athletes: A Tokyo 2020 survey
Source: Temperature (Austin). 2022 Nov 17;10(2):264–75. doi: 10.1080/23328940.2022.2147364 (PMC10274519; doi:10.1080/23328940.2022.2147364)
Supplement: Supplemental Material [file KTMP_A_2147364_SM1769.zip › supplementary/Alkemade - ParalympicQ v1.0submitTemp - supplemental file 1.pdf]

Supplemental file 1

Pre- and post-Paralympic surveys

Article title

Heat preparedness and exertional heat illness in Paralympic athletes: A Tokyo 2020 survey

Authors

Puck Alkemade[1], Hein A. M. Daanen[1], Thomas W. J. Janssen[1], Elizabeth Broad[2], Victoria L. Goosey-Tolfrey[3], Tatsuru Ibusuki[4], Hiske Kneepkens[5], Julien D. Périard[6], Thijs M. H. Eijsvogels[7]

Corresponding author

Puck Alkemade, puckalkemade@gmail.com

Author affiliations

[1]Faculty of Behavioural and Movement Sciences, Vrije Universiteit Amsterdam, Amsterdam Movement Sciences, Amsterdam, The Netherlands.
[2]Freelance Sports Dietitian, Huskisson, Australia.
[3]Peter Harrison Centre for Disability Sport, School of Sport, Exercise and Health Sciences, Loughborough University, Loughborough, Leicestershire LE11 3TU, UK.
[4]Department of Rehabilitation Medicine, Akeno Central Hospital, Oita, Japan.
[5]Sport Medisch Centrum Papendal, NOC*NSF, Arnhem, The Netherlands.
[6]University of Canberra Research Institute for Sport and Exercise, Bruce, Australia.
[7]Radboud Institute for Health Sciences, Department of Physiology, Radboud University Medical Center, Nijmegen, The Netherlands.

Pre-Paralympic survey

Personal details

First name: _______________

Last name: _______________

Date of birth:

Month	Day	Year
dropdown	*dropdown*	*dropdown*

Nationality: _______________

Height (please select the appropriate unit and fill in your height):
Without prostheses/braces

- ○ Centimeters __________________
- ○ Feet-inches __________________
- ○ I don't know

Weight (please select the appropriate unit and fill in your weight):
Without prostheses

- ○ Kilogram _________________
- ○ Pounds __________________
- ○ Stone-pounds_____________
- ○ I don't know

Gender:

- ○ Male
- ○ Female
- ○ Prefer not to say

Impairment

Please specify the health condition that causes your impairment:

Impaired muscle power

- ○ Spinal cord injury
- ○ Muscular dystrophy
- ○ Brachial plexus injury
- ○ Erb's palsy
- ○ Polio
- ○ Spina bifida
- ○ Guillain-Barré syndrome

Impaired passive range of motion (PROM)

- ○ Arthrogryposis
- ○ Ankylosis
- ○ Post burns joint contractures

Limb deficiency

- ○ Amputation resulting from trauma
- ○ Congenital limb deficiency (present at birth)

Leg length difference

- ○ Congenital leg length difference (present at birth)
- ○ Leg length difference resulting from trauma

Short stature

- ○ Achondroplasia
- ○ Growth dysfunction

Hypertonia / Ataxia / Athetosis

- ○ Cerebral palsy
- ○ Stroke
- ○ Brain injury
- ○ Multiple sclerosis
- ○ Friedreich's ataxia
- ○ Spinocerebellar ataxia

Vision impairment

- ○ Myopia
- ○ Tunnel vision
- ○ Scotoma
- ○ Retinitis pigmentosa
- ○ Glaucoma
- ○ Congenital cataract
- ○ Macular degeneration

Intellectual impairment

- ○ Fragile X Syndrome
- ○ Down Syndrome
- ○ Autism Spectrum Disorders

○ Intellectual impairment resulting from trauma
○ Learning deficiency
○ Intellectual retardation

Other (if you did not find your impairment above)
○ Other, please specify: ____________________

Display This Question:
If Please specify the health condition that causes your impairment: = Spinal cord injury

Please specify the range that applies to the level of the spinal cord lesion (tick all that apply):
☐ Cervical (C1-C8)
☐ High thoracic (T1-T6)
☐ Low thoracic (T7-T12)
☐ Lumbar (L1-L5)
☐ Sacral (S1-S5)

Please specify the completeness of the spinal cord lesion:
○ Complete loss of both sensory and motor function below lesion level (ASIA A)
○ Sensory function preserved, but complete loss of motor function below lesion level (ASIA B)
○ Motor function preserved, but complete loss of sensory function below lesion level (ASIA C/D)

How would you rate your ability to sweat below the lesion level, on a scale from 1 to 5?

No sweating response below lesion level				Full sweating response below lesion level
1	2	3	4	5

Display This Question:
If Please specify the health condition that causes your impairment: = Amputation resulting from trauma
Or = Congenital limb deficiency (present at birth)

Please tick the boxes that apply to the limb deficiency:
○ Single upper limb deficiency
○ Bilateral upper limb deficiency
○ Single lower limb deficiency
○ Bilateral lower limb deficiency

Time since impairment:

- 3 years or less
- 3-5 years
- 6-10 years
- More than 10 years
- Congenital (present at birth)

Sports participation

For how many years have you participated in Paralympic sports at the international level?

- 5 years or less
- 6-10 years
- 11-20 years
- 21-30 years
- More than 30 years

Over the last year, what were your average training hours per week? Please slide the pin on the bar to the appropriate number.

	0	4	8	12	16	20	24	28	32	36	40
Average training hours per week											

- I don't know

What competition(s) are you participating in at the Tokyo Paralympics? Tick all that apply.

- ☐ Archery
- ☐ Athletics
- ☐ Badminton
- ☐ Boccia
- ☐ Canoe
- ☐ Cycling
- ☐ Equestrian
- ☐ Football 5-a-side
- ☐ Goalball
- ☐ Judo
- ☐ Powerlifting
- ☐ Rowing
- ☐ Shooting
- ☐ Sitting volleyball
- ☐ Swimming
- ☐ Table tennis
- ☐ Taekwondo
- ☐ Triathlon
- ☐ Wheelchair basketball
- ☐ Wheelchair fencing
- ☐ Wheelchair rugby
- ☐ Wheelchair tennis

Display This Question:
If What competition(s) are you participating in at the Tokyo Paralympics? Tick all that apply. = Athletics

Athletics
Please specify the event(s) that you are participating in during the Tokyo Paralympics. Tick all that apply.

- ☐ Track - 100m
- ☐ Track - 200m
- ☐ Track - 400m
- ☐ Track - 800m
- ☐ Track - 1500m
- ☐ Track - 5000m
- ☐ Track - universal relay (4x100m)
- ☐ Road - marathon
- ☐ Field - high jump
- ☐ Field - long jump
- ☐ Field - discus
- ☐ Field - shot put
- ☐ Field - javelin
- ☐ Field - club throw

Display This Question:
If What competition(s) are you participating in at the Tokyo Paralympics? Tick all that apply. = Cycling

Cycling
Please specify the event(s) you are participating in during the Tokyo Paralympics. Tick all that apply.

- ☐ Road - Road Race
- ☐ Road - Time Trial
- ☐ Road - Team Relay
- ☐ Track - 500m Time Trial
- ☐ Track - 1000m Time Trial
- ☐ Track - Individual Pursuit
- ☐ Track - 750m Team Sprint

Cycling
Please specify your classification:

- ☐ C1
- ☐ C2
- ☐ C3
- ☐ C4
- ☐ C5
- ☐ H1
- ☐ H2
- ☐ H3
- ☐ H4
- ☐ H5
- ☐ T1
- ☐ T2
- ☐ B1
- ☐ B2
- ☐ B3

Display This Question:
If What competition(s) are you participating in at the Tokyo Paralympics? Tick all that apply. = Swimming

Swimming
Please specify the events that you are participating in during the Tokyo Paralympics. Tick all that apply.

- ☐ 50m Freestyle
- ☐ 100m Freestyle
- ☐ 200m Freestyle
- ☐ 400m Freestyle
- ☐ 50m Backstroke
- ☐ 100m Backstroke
- ☐ 50m Breaststroke
- ☐ 100m Breaststroke
- ☐ 50m Butterfly
- ☐ 100m Butterfly
- ☐ 150m Individual Medley
- ☐ 200m Individual Medley
- ☐ 4x100m Freestyle Relay
- ☐ 4x100m Medley Relay
- ☐ 4x50m Freestyle Relay

Display This Question:
If What competition(s) are you participating in at the Tokyo Paralympics? Tick all that apply. = Wheelchair basketball

Wheelchair basketball
Please specify your point value:

- ○ 1.0
- ○ 2.0
- ○ 3.0
- ○ 4.0
- ○ 4.5

Display This Question:
If What competition(s) are you participating in at the Tokyo Paralympics? Tick all that apply. = Wheelchair rugby

Wheelchair rugby
Please specify your point value:
For females, please select your actual classification value (i.e. do not subtract 0.5).

- ○ 0.5
- ○ 1.0
- ○ 1.5
- ○ 2.0
- ○ 2.5
- ○ 3.0
- ○ 3.5

Heat

Have you ever experienced the following heat-stress related symptoms during or immediately (<30 min) after a training or competition? Please answer for each environmental condition.

	Cool (<15 °C / <59 °F)			Neutral (15-25 °C / 59-77 °F)			Hot (>25 °C / >77 °F)		
	Yes	No	Not applicable	Yes	No	Not applicable	Yes	No	Not applicable
Cramping	○	○	○	○	○	○	○	○	○
Nausea	○	○	○	○	○	○	○	○	○
Vomiting	○	○	○	○	○	○	○	○	○
Dizziness	○	○	○	○	○	○	○	○	○
Severe headache	○	○	○	○	○	○	○	○	○
Collapsing / fainting	○	○	○	○	○	○	○	○	○
Other (please specify)	○	○	○	○	○	○	○	○	○

Have you ever been diagnosed with exertional heat illness?

"Diagnosed" means that this was determined by first-aid personnel, your team physician, general practitioner or in a hospital. Examples: dehydration, hyponatremia, heat syncope, heat exhaustion, heat stroke.

- ○ Yes
- ○ No

Display This Question:
If Have you ever been diagnosed with exertional heat illness? = Yes

What diagnoses did you receive, and how many times did you receive a diagnosis?
The diagnoses should have been made by first-aid personnel, your team physician, general practitioner or in a hospital.

	**What diagnoses did you receive?**	**How many times did you receive this diagnosis?**
	Tick all that apply	
Dehydration<br>*Severe fluid deficit in the body*	☐	*Dropdown*<br>*1 – More than 5*
Hyponatremia<br>*Low sodium concentration in the blood*	☐	*Dropdown*<br>*1 – More than 5*
Heat syncope<br>*Fainting as a result of overheating*	☐	*Dropdown*<br>*1 – More than 5*
Heat exhaustion<br>*Inability to effectively exercise due to elevated body core temperature.*	☐	*Dropdown*<br>*1 – More than 5*
Heat stroke<br>*Most severe heat illness. Collapse/confusion/ seizures due to high body core temperature.*	☐	*Dropdown*<br>*1 – More than 5*
I don't know	☐	*Dropdown*<br>*1 – More than 5*

Display This Question:
If Have you ever been diagnosed with exertional heat illness? = Yes

How long ago did you receive the last diagnosis?
The diagnosis should have been made by first-aid personnel, your team physician, general practitioner or in a hospital.

- ○ In the past year
- ○ 1-3 years ago
- ○ 3-5 years ago
- ○ 5-10 years ago
- ○ 10-20 years ago
- ○ More than 20 years ago

How would you rate your own capacity to handle exercise in warm/hot conditions (>25°C / 77°F)?

- ○ Very good
- ○ Good
- ○ Average
- ○ Bad
- ○ Very bad

Preparatory strategies: Heat acclimation

In the last year, how much time in total have you spent on a location with a hot climate (>25°C / 77°F)?

If you have been on multiple trips to locations with a hot climate, please add up the total time of these trips.

- ○ None
- ○ 0-2 months
- ○ 2-4 months
- ○ 4-6 months
- ○ 6-8 months
- ○ 8-12 months

“Heat acclimation” is an effective strategy to prepare for competition in warm/hot environments. To “acclimate” (i.e. adapt) to the heat, athletes have to expose themselves to a warm/hot environment on multiple occasions. This can, for example, be done in a naturally hot environment, climatic chamber, or using a hot bath or sauna.

Have you ever used a heat acclimation strategy in preparation for competition before (excluding preparation Tokyo Paralympics)?

- ○ Yes
- ○ No

Display This Question:
If Have you ever used a heat acclimation strategy in preparation for competition before? = Yes

What heat acclimation strategies have you ever used before (excluding preparation for Tokyo Paralympics)? Tick all that apply.

- ☐ Training in a natural hot environment (e.g. training camp)
- ☐ Training in an artificial hot room / laboratory / climate chamber
- ☐ Sauna bathing
- ☐ Hot water bathing
- ☐ Other (please specify) ____________________

Display This Question:
If Have you ever used a heat acclimation strategy in preparation for competition before? = No

Why have you never used a heat acclimations strategy before (excluding preparation for Tokyo Paralympics)? Tick all that apply.

- ☐ I have never competed in warm/hot conditions before
- ☐ I didn't have the financial resources
- ☐ I and/or my support team did not have sufficient knowledge about this topic
- ☐ It did not fit in my training schedule
- ☐ I thought it was not effective
- ☐ Other (please specify) ________________________

Did/will you use a heat acclimation strategy in preparation for the Tokyo Paralympics?

- ○ Yes
- ○ No

Display This Question:
If Did/will you use a heat acclimation strategy in preparation for the Tokyo Paralympics? = Yes

What heat acclimation strategy did/will you use in preparation for the Tokyo Paralympics? Tick all that apply.

- ☐ Training in a natural hot environment (e.g. training camp)
- ☐ Training in an artificial hot room / laboratory / climate chamber
- ☐ Sauna bathing
- ☐ Hot water bathing
- ☐ Other (please specify) ____________________

Please specify the following characteristics of your heat acclimation strategy for the Tokyo Paralympics:

	0	5	10	15	20	25	30
How many heat acclimation sessions did/will you complete in total?							
Over how many days were/are these sessions distributed?							

Display This Question:
If Did/will you use a heat acclimation strategy in preparation for the Tokyo Paralympics? = No

Why did/will you not use a heat acclimation strategy in preparation for the Tokyo Paralympics? Tick all that apply.

- ☐ I don't have the financial resources
- ☐ I and/or my support team do(es) not have sufficient knowledge about this topic
- ☐ It does not fit in my training schedule
- ☐ I think it is not effective
- ☐ I think it is not needed for this event
- ☐ Other (please specify) ________________________

When will you arrive in Tokyo or surroundings for the Paralympics?
If you currently live in Tokyo, please select "Not applicable" in both categories

Month	Day
dropdown	*dropdown*

When will you have your first match/race of the Tokyo Paralympics?

Month	Day
dropdown	*dropdown*

Preparatory strategies: Cooling

Have you ever used cooling method(s) before or during competition (excluding preparation for Tokyo Paralympics)?

- ○ Yes
- ○ No

Display This Question:
If Have you ever used cooling method(s) before or during competition? = Yes

Please tick all cooling methods that you have ever used before and during (a) race(s)/match(es):

- *Cooling before exercise is called pre-cooling, meaning cooling prior to the exercise.*
- *Cooling during exercise is called per-cooling, meaning cooling during the exercise.*
  This includes cooling during breaks (for example, when subbing or during halftime).

	**Before (a) race(s)/match(es)** ***i.e. pre-cooling***	**During (a) race(s)/match(es)** ***i.e. per-cooling***
	Tick all that apply	Tick all that apply
Cold towel(s)	○	○
Neck collar	○	○
Ice bag(s)	○	○
Water spray on the face and/or neck	○	○
Water spray on the torso, arms and/or legs	○	○
Fanning	○	○
Evaporation vest (submerged in water)	○	○
Ice vest / PCM vest	○	○
Whole-body cold water immersion	○	○
Cold water immersion of the hands	○	○
Cold water immersion of the legs	○	○
Cold water immersion of the feet	○	○
Cold beverage ingestion	○	○
Ice-slurry ingestion	○	○
Menthol mouth rinse	○	○
Ethanol/menthol on the skin of the face and/or neck	○	○
Ethanol/menthol on the skin of the torso, arms and/or legs	○	○
Other (please specify) ________________________	○	○

Display This Question:
If Have you ever used cooling method(s) before or during competition? = No

Why have you never used cooling method(s) before or during competition (excluding preparation for Tokyo Paralympics)? Tick all that apply.

- ☐ I have never competed in warm/hot conditions
- ☐ I didn't have the financial resources
- ☐ I and/or my support team did not have sufficient knowledge about this topic
- ☐ I thought it was not effective
- ☐ I didn't like the cooling
- ☐ Other (please specify) ________________________
  ____

Will you use cooling method(s) during the Tokyo Paralympics?

- ○ Yes
- ○ No

Display This Question:
If Will you use cooling method(s) during the Tokyo Paralympics? = Yes

What cooling method(s) do you plan on using before and during the race(s)/match(es) at the Tokyo Paralympics? Tick all that apply.

- *Cooling before exercise is called pre-cooling, meaning cooling prior to the exercise.*
- *Cooling during exercise is called per-cooling, meaning cooling during the exercise. This includes cooling during breaks (for example, when subbing or during halftime).*

	**Before (a) race(s)/match(es) *i.e. pre-cooling***	**During (a) race(s)/match(es) *i.e. per-cooling***
	Tick all that apply	Tick all that apply
Cold towel(s)	○	○
Neck collar	○	○
Ice bag(s)	○	○
Water spray on the face and/or neck	○	○
Water spray on the torso, arms and/or legs	○	○
Fanning	○	○
Evaporation vest (submerged in water)	○	○
Ice vest / PCM vest	○	○

Whole-body cold water immersion	○	○
Cold water immersion of the hands	○	○
Cold water immersion of the legs	○	○
Cold water immersion of the feet	○	○
Cold beverage ingestion	○	○
Ice-slurry ingestion	○	○
Menthol mouth rinse	○	○
Ethanol/menthol on the skin of the face and/or neck	○	○
Ethanol/menthol on the skin of the torso, arms and/or legs	○	○
Other (please specify) ________________	○	○

Display This Question:
If Will you use cooling method(s) during the Tokyo Paralympics? = No

Why will you not use cooling method(s) during the Tokyo Paralympics? Tick all that apply.

- ▢ I don't have the financial resources
- ▢ I and/or my support team do(es) not have sufficient knowledge about this topic
- ▢ I think it is not effective
- ▢ I don't like the cooling
- ▢ I think it is not needed for this event
- ▢ Other (please specify) ________________

Preparatory strategies: Hydration

Do you have a hydration plan for the hours leading up to your race(s) or match(es) in the Tokyo Paralympics?
i.e. do you plan in advance how much and/or what you will drink?

- ○ Yes
- ○ No

Display This Question:
If Do you have a hydration plan for the hours leading up to your race(s) or match(es) in the Tokyo Paralympics? = Yes

Please specify what the fluid(s) in your hydration plan include(s). Tick all boxes that apply.
This question concerns the hydration plan for the hours leading up to your race(s) or match(es) in the Tokyo Paralympics.

- ☐ Water
- ☐ Electrolytes (examples: salt, magnesium, sodium, potassium, chloride and calcium)
- ☐ Carbohydrates/sugars
- ☐ Glycerol
- ☐ Creatine
- ☐ Ice-slurry
- ☐ Other (please specify) ________________________

____

Do you have a hydration plan for during your race(s) or match(es) in the Tokyo Paralympics?

- ○ Yes
- ○ No
- ○ Not applicable

Display This Question:
If Do you have a hydration plan for during your race(s) or match(es) in the Tokyo Paralympics? = Yes

Please specify what the fluid(s) in your hydration plan include(s). Tick all boxes that apply.
This question concerns the hydration plan during your race(s) or match(es) in the Tokyo Paralympics.

- ☐ Water
- ☐ Electrolytes (examples: salt, magnesium, sodium, potassium, chloride and calcium)
- ☐ Carbohydrates/sugars
- ☐ Glycerol
- ☐ Creatine
- ☐ Ice-slurry
- ☐ Other (please specify) ________________________

____

Preparatory strategies: Support

Display This Question:
If Did/will you use a heat acclimation strategy in preparation for the Tokyo Paralympics? = Yes
Or Will you use cooling method(s) during the Tokyo Paralympics? = Yes
Or Do you have a hydration plan for the hours leading up to… Tokyo Paralympics = Yes
Or Do you have a hydration plan for during your race(s) or match(es) in the Tokyo Paralympics? = Yes

You indicated that you will use a heat acclimation strategy, cooling and/or hydration plan for the Tokyo Paralympics. What support did you receive in developing these strategies? Tick all that apply.

- ☐ Support of a sport scientist, dietitian and/or physician
- ☐ Support of my coach
- ☐ Support of other athletes
- ☐ No external support (I decided myself)
- ☐ Other (please specify) ____________________

What factors had an influence on the development of your heat acclimation / cooling / hydration strategies? Tick all that apply.

- ☐ Previous experiences with these strategies
- ☐ Recent research findings
- ☐ Strategies of other athletes/competitors
- ☐ Ability to match with regular training program
- ☐ Financial resources
- ☐ Limitations due to COVID-19 regulations
- ☐ I don't know, the strategies were developed for me
- ☐ Other (please specify) __________________
- ☐ None of the above

Post-Paralympic survey

On average, how comfortable were you with the climate during your races/matches at the 2020 Paralympics? Please provide a rating on a scale from 1 to 10 (0 = Extremely uncomfortable / 10 = Extremely comfortable).

0 = Extremely uncomfortable | 5 = Neutral | 10 = Extremely comfortable

0 1 2 3 4 5 6 7 8 9 10

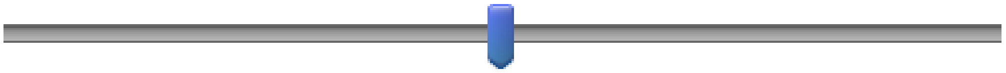

Did you experience any of the following heat-related illness symptoms **<u>during or immediately (<30 min) after</u>** any of your races/matches at the Tokyo Paralympics? Tick all that apply. Please also select in how many races/matches you had these symptoms.

	**Did you experience any of these symptoms?**	**In how many races/matches did you have these symptoms?**
	Tick all that apply	
Cramping	☐	*Dropdown 1 – More than 5*
Nausea	☐	*Dropdown 1 – More than 5*
Vomiting	☐	*Dropdown 1 – More than 5*
Dizziness	☐	*Dropdown 1 – More than 5*
Severe headache	☐	*Dropdown 1 – More than 5*
Collapsing / fainting	☐	*Dropdown 1 – More than 5*
Other (please specify)	☐	*Dropdown 1 – More than 5*

Were you diagnosed with exertional heat illness during the Tokyo 2020 Paralympics?
"Diagnosed" means that this was determined by first-aid personnel, your team physician, general practitioner or in a hospital.
Examples: dehydration, hyponatremia, heat syncope, heat exhaustion, heat stroke.

- ○ Yes
- ○ No

Display This Question:
If Were you diagnosed with exertional heat illness during the Tokyo 2020 Paralympics? = Yes

What diagnoses did you receive?
The diagnoses should have been made by first-aid personnel, your team physician, general practitioner or in a hospital.

- ☐ Dehydration
  Severe fluid deficit in the body
- ☐ Hyponatremia
  Low sodium concentration in the blood
- ☐ Heat syncope
  Fainting as a result of overheating
- ☐ Heat exhaustion
  Inability to effectively exercise due to elevated body core temperature.
- ☐ Heat stroke
  Most severe heat illness. Collapse/confusion/ seizures due to high body core temperature.
- ☐ I don't know

“Heat acclimation” is an effective strategy to prepare for competition in warm/hot environments. To “acclimate” (i.e. adapt) to the heat, athletes have to expose themselves to a warm/hot environment on multiple occasions. This can, for example, be done in a naturally hot environment, climatic chamber, or using a hot bath or sauna.

Did you use heat acclimation in preparation for the 2020 Tokyo Paralympics?

- ○ Yes
- ○ No

Display This Question:

If Did you use heat acclimation in preparation for the 2020 Tokyo Paralympics? = Yes

Did your heat acclimation strategy go according to your plans made before the 2020 Tokyo Paralympics?

- ○ Yes, it went according to plan
- ○ No, it did not go according to plan

Display This Question:

If Did your heat acclimation strategy go according to your plans made before the 2020 Tokyo Paralympics? = No, it did not go according to plan.

Why did your heat acclimation strategy not go according to plan?
For example:
Did you not complete the planned amount of heat acclimation days?
Was the ambient temperature during heat acclimation not as expected?

Did you use cooling strategies before or during (a) race(s)/match(es) at the 2020 Paralympics?
For example: cold towels, water spray, ice slushy, menthol

- ○ Yes
- ○ No

Display This Question:

If Did you use cooling strategies before or during (a) race(s)/match(es) at the 2020 Paralympics = Yes

Please tick all cooling methods that you have used before and during (a) race(s)/match(es) at the 2020 Paralympics:

- *Cooling before exercise is called pre-cooling, meaning cooling prior to the exercise.*
- *Cooling during exercise is called per-cooling, meaning cooling during the exercise. This includes cooling during breaks (for example, when subbing or during halftime).*

	**Before (a) race(s)/match(es) *i.e. pre-cooling***	**During (a) race(s)/match(es) *i.e. per-cooling***
	Tick all that apply	Tick all that apply
Cold towel(s)	○	○
Neck collar	○	○
Ice bag(s)	○	○
Water spray on the face and/or neck	○	○
Water spray on the torso, arms and/or legs	○	○
Fanning	○	○
Evaporation vest (submerged in water)	○	○
Ice vest / PCM vest	○	○
Whole-body cold water immersion	○	○
Cold water immersion of the hands	○	○
Cold water immersion of the legs	○	○
Cold water immersion of the feet	○	○
Cold beverage ingestion	○	○
Ice-slurry ingestion	○	○
Menthol mouth rinse	○	○
Ethanol/menthol on the skin of the face and/or neck	○	○
Ethanol/menthol on the skin of the torso, arms and/or legs	○	○
Other (please specify) ________________________	○	○

Display This Question:

If Did you use cooling strategies before or during (a) race(s)/match(es) at the 2020 Paralympics = Yes

Did you use cooling strategies according to your plans made before the 2020 Tokyo Paralympics?

- ○ Yes, it went according to plan
- ○ No, it did not go according to plan

Display This Question:

If Did you use cooling strategies according to your plans made before the 2020 Tokyo Paralympics? = No, it did not go according to plan.

Why did your cooling strategies not go according to plan?
For example:
Did you have access to your planned cooling strategies?
Did you need (more) cooling?

How satisfied were you with your heat preparedness for the 2020 Paralympics? Please provide a score for both your heat acclimation strategy and cooling strategy, on a scale from 1 to 10 (0 = Not satisfied at all / 10 = Perfectly satisfied).

Note: If you did not use heat acclimation or cooling, select "not applicable"

	Not satisfied at all										Perfectly satisfied	Not Applicable
	0	1	2	3	4	5	6	7	8	9	10	
Heat acclimation strategy												○
Cooling strategy/ strategies												○

Please briefly explain the rating you gave for heat acclimation. Why were you (not) satisfied?

Please briefly explain the rating you gave for cooling. Why were you (not) satisfied?
