# Supplementary material for: Heat preparedness and exertional heat illness in Paralympic athletes: A Tokyo 2020 survey
Source: Temperature (Austin). 2022 Nov 17;10(2):264–75. doi: 10.1080/23328940.2022.2147364 (PMC10274519; doi:10.1080/23328940.2022.2147364)
Supplement: Supplemental Material [file KTMP_A_2147364_SM1769.zip › supplementary/Alkemade - ParalympicQ v1.0submitTemp - supplemental file 2.pdf]

Supplemental file 2

Variable grouping (impairments, sports and cooling strategies)

Article title

Heat preparedness and exertional heat illness in Paralympic athletes: A Tokyo 2020 survey

Authors

Puck Alkemade[1], Hein A. M. Daanen[1], Thomas W. J. Janssen[1], Elizabeth Broad[2], Victoria L. Goosey-Tolfrey[3], Tatsuru Ibusuki[4], Hiske Kneepkens[5], Julien D. Périard[6], Thijs M. H. Eijsvogels[7]

Corresponding author

Puck Alkemade, puckalkemade@gmail.com

Author affiliations

[1]Faculty of Behavioural and Movement Sciences, Vrije Universiteit Amsterdam, Amsterdam Movement Sciences, Amsterdam, The Netherlands.
[2]Freelance Sports Dietitian, Huskisson, Australia.
[3]Peter Harrison Centre for Disability Sport, School of Sport, Exercise and Health Sciences, Loughborough University, Loughborough, Leicestershire LE11 3TU, UK.
[4]Department of Rehabilitation Medicine, Akeno Central Hospital, Oita, Japan.
[5]Sport Medisch Centrum Papendal, NOC*NSF, Arnhem, The Netherlands.
[6]University of Canberra Research Institute for Sport and Exercise, Bruce, Australia.
[7]Radboud Institute for Health Sciences, Department of Physiology, Radboud University Medical Center, Nijmegen, The Netherlands.

Table 1 Impairments of respondents, including categories.

**Impairment**▶	**n**	**Category**
Other (please specify)	30	Multiple
Spinal cord injury	17	SCI
Amputation resulting from trauma	13	Limb deficiency
Congenital limb deficiency (present at birth)	12	Limb deficiency
Intellectual retardation	7	Visual/Intellectual/Pilot
Brain injury	3	Other
Stroke	3	Other
Arthrogryposis	2	Other
Brachial plexus injury	2	Other
Cerebral palsy	2	Other
Macular degeneration	2	Visual/Intellectual/Pilot
Multiple sclerosis	2	Other
Tunnel vision	2	Visual/Intellectual/Pilot
Ankylosis	1	Other
Autism Spectrum Disorders	1	Visual/Intellectual/Pilot
Congenital cataract	1	Visual/Intellectual/Pilot
Guillain-Barré syndrome	1	Other
Muscular dystrophy	1	Other
Myopia	1	Visual/Intellectual/Pilot
Polio	1	Other
Retinitis pigmentosa	1	Visual/Intellectual/Pilot
Spina bifida	1	SCI

▶Impairments selected from multiple-choice list

Table 2 Sports performed by respondents, including environment and category, and specific disciplines where appropriate.

**Sports**$^{\alpha}$	**n**	**Environment**	**Category**	***Disciplines***
Athletics	29	Outdoor	Endurance (n=11)<br>Power (n=18)	*100m (n=8); 1500m (n=8); Long jump (n=7); Marathon (n=5); 400m (n=6); Javelin (n=3); 200m (n=3); 5000m (n=3); Universal relay 4x100m (n=2); Club throw (n=1); 800m (n=1).*
Swimming	16	Indoor	Power	*Distances (various strokes): 100m (n=23); 200m (n=8); 50m (n=6); 400m (n=4).*
Wheelchair basketball	11	Indoor	Mixed	
Cycling	8	Indoor (n=1)<br>Outdoor (n=7)	Endurance (n=7)<br>Power (n=1)	*Road race (n=7); Road time trial (n=7); Track 500m time trial (n=2); Track individual pursuit (n=2).*
Rowing	7	Outdoor	Endurance	
Sitting volleyball	6	Indoor	Mixed	
Equestrian	4	Outdoor	Skill	
Wheelchair tennis	4	Outdoor$^{¥}$	Mixed	
Archery	3	Outdoor	Skill	
Football 5-a-side	3	Outdoor	Mixed	
Judo	3	Indoor	Power	
Canoe	2	Outdoor	Power	
Triathlon	2	Outdoor	Endurance	
Wheelchair rugby	2	Indoor	Mixed	
Athletics, Triathlon	1	Outdoor	Endurance	*Athletics: marathon (n=1).*
Cycling, Triathlon	1	Outdoor	Endurance	*Cycling: road race (n=1), time trial (n=1).*
Powerlifting	1	Indoor	Power	
Shooting	1	Outdoor	Skill	
Table tennis	1	Indoor	Skill	
Taekwondo	1	Indoor	Power	
Wheelchair fencing	1	Indoor	Mixed	

$^{\alpha}$Sports performed by respondents (n), with two respondents competing in two sports. $^{¥}$Tennis was played both indoor and outdoor; when WBGT limits were exceeded, matches on Center Court were played with retracted roof.

Table 3 Sex, continent and impairment of indoor and outdoor athletes.

		Indoor	Outdoor
Sex	Male	15 (29%)	36 (71%)
	Female	28 (50%)	28 (50%)
Continent	Africa	1 (50%)	1 (50%)
	Asia	12 (29%)	30 (71%)
	Europe	14 (36%)	25 (64%)
	North-America	6 (86%)	1 (14%)
	Oceania	3 (30%)	7 (70%)
	South-America	7 (100%)	0 (0%)
Impairment	SCI	6 (32%)	13 (68%)
	Limb deficiency	11 (41%)	16 (59%)
	Visual/Intellectual/None	7 (30%)	16 (70%)
	Other	19 (51%)	18 (49%)

Table 4 Cooling strategy questions strategies included 18 options (incl. "Other" to be specified), but strategies were later categorized into 7 main categories. A cooling category was selected when the participant used one or multiple strategies in that category.

Category	Strategies
Beverage	Ice-slurry ingestion<br>Cold beverage ingestion
Cold towels/bags	Cold towel(s)<br>Neck collar<br>Ice bag(s)
Cold water immersion	Whole-body cold water immersion<br>Cold water immersion of the hands<br>Cold water immersion of the legs<br>Cold water immersion of the feet
Cooling vest	Cold towel(s)<br>Neck collar<br>Ice bag(s)
Fanning	Fanning
Menthol	Menthol mouth rinse<br>Ethanol/menthol on the skin of the face and/or neck<br>Ethanol/menthol on the skin of the torso, arms and/or legs
Water spray	Water spray on the face and/or neck<br>Water spray on the torso, arms and/or legs
