# Supplementary material for: Heat preparedness and exertional heat illness in Paralympic athletes: A Tokyo 2020 survey
Source: Temperature (Austin). 2022 Nov 17;10(2):264–75. doi: 10.1080/23328940.2022.2147364 (PMC10274519; doi:10.1080/23328940.2022.2147364)
Supplement: Supplemental Material [file KTMP_A_2147364_SM1769.zip › supplementary/Alkemade - ParalympicQ v1.0submitTemp - supplemental file 3.pdf]

Supplemental file 3

Hydration plans and strategy development in Tokyo

Article title

Heat preparedness and exertional heat illness in Paralympic athletes: A Tokyo 2020 survey

Authors

Puck Alkemade[1], Hein A. M. Daanen[1], Thomas W. J. Janssen[1], Elizabeth Broad[2], Victoria L. Goosey-Tolfrey[3], Tatsuru Ibusuki[4], Hiske Kneepkens[5], Julien D. Périard[6], Thijs M. H. Eijsvogels[7]

Corresponding author

Puck Alkemade, puckalkemade@gmail.com

Author affiliations

[1]Faculty of Behavioural and Movement Sciences, Vrije Universiteit Amsterdam, Amsterdam Movement Sciences, Amsterdam, The Netherlands.
[2]Freelance Sports Dietitian, Huskisson, Australia.
[3]Peter Harrison Centre for Disability Sport, School of Sport, Exercise and Health Sciences, Loughborough University, Loughborough, Leicestershire LE11 3TU, UK.
[4]Department of Rehabilitation Medicine, Akeno Central Hospital, Oita, Japan.
[5]Sport Medisch Centrum Papendal, NOC*NSF, Arnhem, The Netherlands.
[6]University of Canberra Research Institute for Sport and Exercise, Bruce, Australia.
[7]Radboud Institute for Health Sciences, Department of Physiology, Radboud University Medical Center, Nijmegen, The Netherlands.

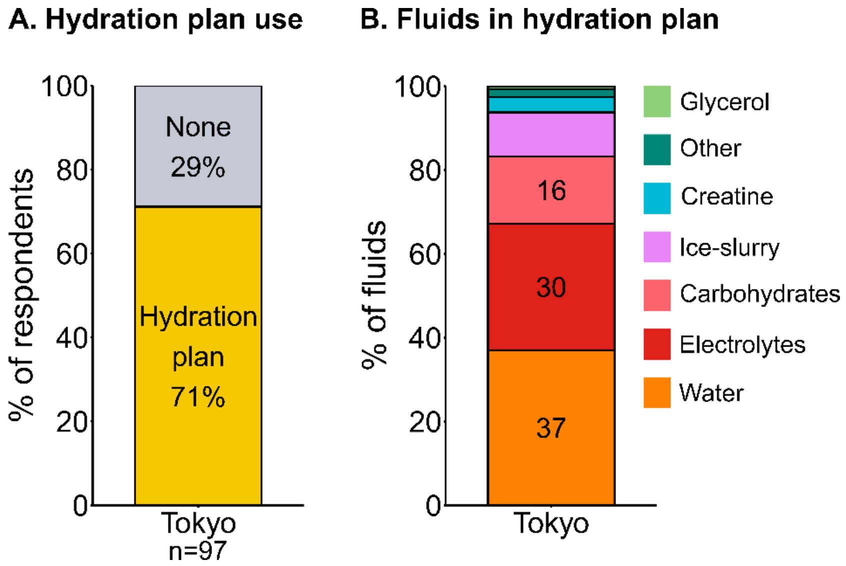

Fig. 1 A; Percentage of respondents with an hydration plan in Tokyo, either pre- or during competition. **B;** Prevalence of fluids used in hydration plans in Tokyo. Percentages >10% are displayed in the appropriate bars.

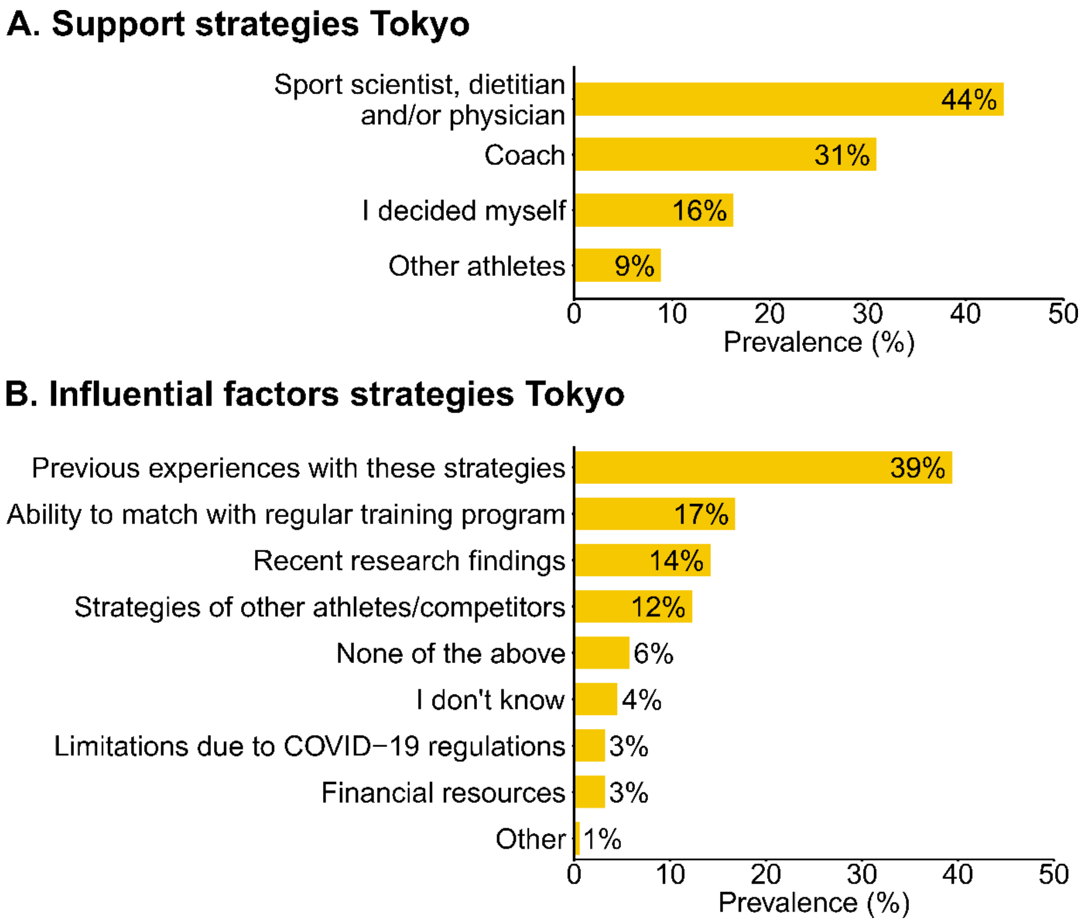

Fig. 2 A; Support received by athletes in developing heat mitigation strategies for the Tokyo 2020 Paralympic Games. **B;** Factors that influenced strategy development for Tokyo. Prevalence is expressed in percentage of total answers selected.
