# Supplementary material for: Heat preparedness and exertional heat illness in Paralympic athletes: A Tokyo 2020 survey
Source: Temperature (Austin). 2022 Nov 17;10(2):264–75. doi: 10.1080/23328940.2022.2147364 (PMC10274519; doi:10.1080/23328940.2022.2147364)
Supplement: Supplemental Material [file KTMP_A_2147364_SM1769.zip › supplementary/Alkemade - ParalympicQ v1.0submitTemp - supplemental file 4.pdf]

Supplemental file 4

Ordinal and binary logistic regression outcomes

Article title

Heat preparedness and exertional heat illness in Paralympic athletes: A Tokyo 2020 survey

Authors

Puck Alkemade[1], Hein A. M. Daanen[1], Thomas W. J. Janssen[1], Elizabeth Broad[2], Victoria L. Goosey-Tolfrey[3], Tatsuru Ibusuki[4], Hiske Kneepkens[5], Julien D. Périard[6], Thijs M. H. Eijsvogels[7]

Corresponding author

Puck Alkemade, puckalkemade@gmail.com

Author affiliations

[1]Faculty of Behavioural and Movement Sciences, Vrije Universiteit Amsterdam, Amsterdam Movement Sciences, Amsterdam, The Netherlands.
[2]Freelance Sports Dietitian, Huskisson, Australia.
[3]Peter Harrison Centre for Disability Sport, School of Sport, Exercise and Health Sciences, Loughborough University, Loughborough, Leicestershire LE11 3TU, UK.
[4]Department of Rehabilitation Medicine, Akeno Central Hospital, Oita, Japan.
[5]Sport Medisch Centrum Papendal, NOC*NSF, Arnhem, The Netherlands.
[6]University of Canberra Research Institute for Sport and Exercise, Bruce, Australia.
[7]Radboud Institute for Health Sciences, Department of Physiology, Radboud University Medical Center, Nijmegen, The Netherlands.

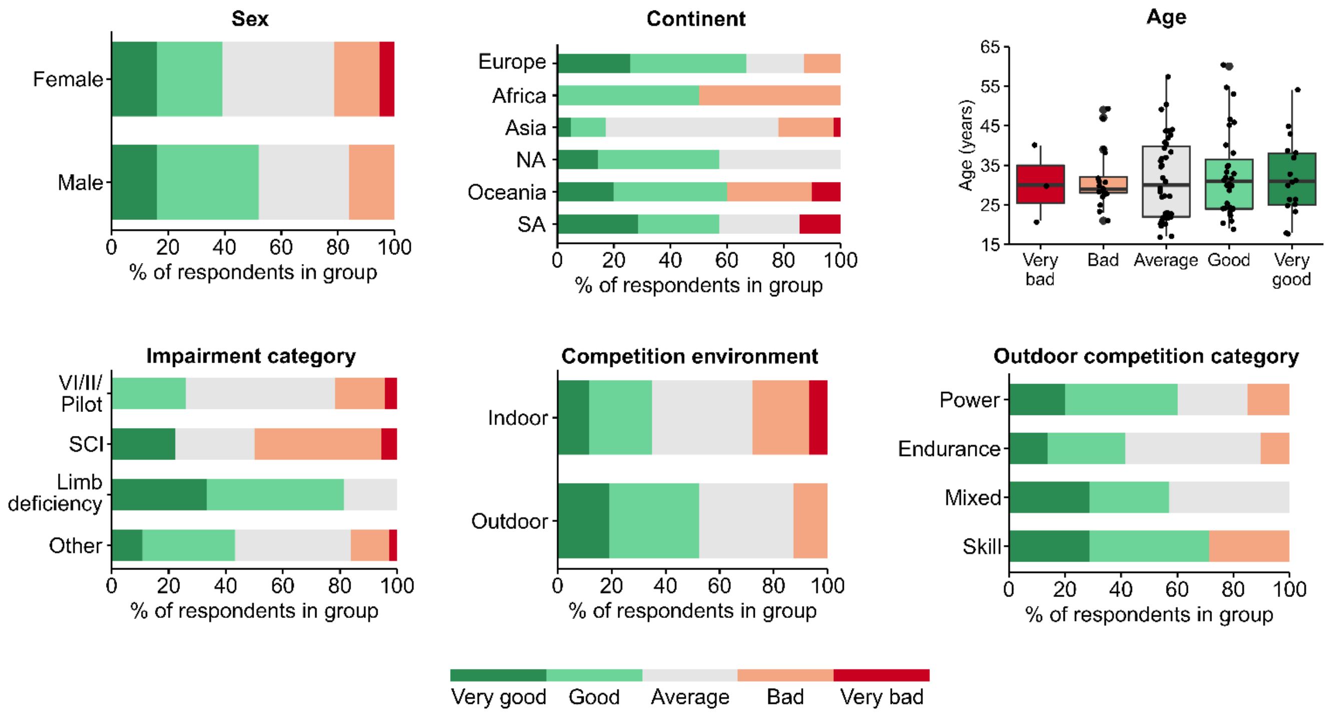

Fig. 1 Self-rated heat-coping ability by participant groups. Abbreviations: NA, North-America; SA, South-America; VI, Visual impairment; II, Intellectual impairment; SCI, spinal cord injury.

Table 1 Explanatory models for self-rated heat-coping ability, with participant characteristics as predictor variables. OR (95% CI), p-value and *n* respondents per group are presented. Abbreviations: OR, odds ratio; CI, confidence interval; Ref, reference group.

	**Self-rated heat coping ability**
**Sex**	***Ref: Female****; n=56*
Male	OR=1.5 (0.7,3); p=0.3; n=50
**Age**	n=106
**Continent**	***Ref: Europe****; n=39*
Africa	OR=0.2 (0,3.4); p=0.3; n=2
Asia	OR=0.2 (0.1,0.5); p<0.001; n=41
North-America	OR=0.8 (0.2,3.1); p=0.7; n=7
Oceania	OR=0.4 (0.1,1.9); p=0.3; n=10
South-America	OR=0.8 (0.2,3.8); p=0.8; n=7
**Impairment category**	***Ref: VI/II/Pilot****; n=23*
Spinal cord injury	OR=0.5 (0.2,1.7); p=0.3; n=18
Limb deficiency	OR=8.9 (3.1,26.5); p<0.001; n=27
Other	OR=1.9 (0.7,5); p=0.18; n=37
**Competition environment**	***Ref: Indoor****; n=43*
Outdoor	OR=2.2 (1.1,4.7); p=0.03; n=63
**Competition category, outdoor**	***Ref: Power****; n=20*
Endurance	OR=0.6 (0.2,1.8); p=0.4; n=29
Mixed	OR=1.4 (0.3,6.7); p=0.7; n=7
Skill	OR=1.3 (0.2,7); p=0.8; n=7

Table 2 Explanatory models for reported heat-related symptom occurrence in the past and in Tokyo, with participant characteristics as predictor variables. Percentage of "Yes"-responders, OR (95% CI), p-value and *n* respondents per group are presented. Abbreviations: OR, odds ratio; CI, confidence interval; Ref, reference group.

	**Heat-related illness symptoms in the past (hot conditions)**	**Heat-related illness symptoms in Tokyo**
**Sex**	***Ref: Female****; 58%. n=45*	***Ref: Female****; 28%. n=29*
Male	60%. OR=1.1 (0.5,2.5); p=0.9; n=47	12%. OR=0.4 (0.1,1.7); p=0.2; n=24
**Age**	OR=1 (0.9, 1.); p=0.6; n = 92	OR=1 (0.9, 1); p=0.2; n=53
**Continent**	***Ref: Europe****; 53%. n=34*	***Ref: Europe****; 19%. n=21*
Africa	50%. OR=0.9 (0,16.3); p=0.9; n=2	-
Asia	62%. OR=1.4 (0.5,3.8); p=0.5; n=34	5%. OR=0.2 (0,2.4); p=0.2; n=19
North-America	71%. OR=2.2 (0.4,13.6); p=0.4; n=7	33%. OR=2.1 (0.1,31.3); p=0.6; n=3
Oceania	78%. OR=3.1 (0.5,17.8); p=0.2; n=9	43%. OR=3.2 (0.5,21.1); p=0.2; n=7
South-America	33%. OR=0.4 (0.1,2.9); p=0.4; n=6	67%. OR=8.5 (0.6,125.2); p=0.1; n=3
**Impairment category**	***Ref: VI/II/Pilot****; 45%. n=20*	***Ref: VI/II/Pilot****; 10%. n=10*
Spinal cord injury	76%. OR=4 (0.9,17); p=0.058; n=17	30%. OR=3.9 (0.3,47.9); p=0.3; n=10
Limb deficiency	46%. OR=1 (0.3,3.5); p>0.9; n=24	0%. OR=0 (0,Inf); p>0.9; n=12
Other	70%. OR=2.9 (0.9,9.5); p=0.1; n=30	35%. OR=4.8 (0.5,48.7); p=0.2; n=20
**Competition environment**	***Ref: Indoor****; 54%. n=37*	***Ref: Indoor****; 30%. n=20*
Outdoor	62%. OR=1.4 (0.6,3.3); p=0.5; n=55	15%. OR=0.4 (0.1,1.7); p=0.2; n=33
**Competition category, outdoor**	***Ref: Power****; 67%. n=18*	***Ref: Power****; 12%. n=8*
Endurance	62%. OR=0.8 (0.2,3.1); p=0.8; n=24	19%. OR=1.6 (0.1,19.5); p=0.7; n=16
Mixed	57%. OR=0.7 (0.1,4.1); p=0.7; n=7	0%. OR=0 (0,Inf); p>0.9; n=5
Skill	50%. OR=0.5 (0.1,3.4); p=0.5; n=6	25%. OR=2.3 (0.1,54.3); p=0.6; n=4

Table 3 Explanatory models for heat acclimation use in the past and in Tokyo, with participant characteristics as predictor variables. Percentage of "Yes"-responders, OR (95% CI), p-value and *n* respondents per group are presented. Abbreviations: OR, odds ratio; CI, confidence interval; Ref, reference group.

	**Heat acclimation use in the past**	**Heat acclimation use in Tokyo**
**Sex**	***Ref: Female****; 35%. n=55*	***Ref: Female****; 57%. n=54*
Male	56%. OR=2.4 (1.1,5.4); p=0.03; n=50	58%. OR=1 (0.5,2.3); p=0.9; n=48
**Age**	OR=1 (1, 1.1); p=0.1; n=105	OR=1 (1, 1.1); p=0.3; n=102
**Continent**	***Ref: Europe****; 46%. n=39*	***Ref: Europe****; 58%. n=36*
Africa	50%. OR=1.2 (0.1,21.2); p=0.9; n=2	50%. OR=0.7 (0,13.1); p=0.8; n=2
Asia	38%. OR=0.7 (0.3,1.7); p=0.4; n=40	55%. OR=0.9 (0.3,2.2); p=0.8; n=40
North-America	43%. OR=0.9 (0.2,4.6); p=0.9; n=7	71%. OR=1.8 (0.3,10.9); p=0.5; n=7
Oceania	90%. OR=10.5 (1.2,95.1); p=0.03; n=10	80%. OR=2.9 (0.5,15.9); p=0.2; n=10
South-America	14%. OR=0.2 (0,1.9); p=0.1; n=7	29%. OR=0.3 (0,1.7); p=0.2; n=7
**Impairment category**	***Ref: VI/II/Pilot****; 45%. n=22*	***Ref: VI/II/Pilot****; 40%. n=20*
Spinal cord injury	44%. OR=1 (0.3,3.4); p=0.9; n=18	61%. OR=2.4 (0.6,8.9); p=0.2; n=18
Limb deficiency	59%. OR=1.7 (0.5,5.6); p=0.3; n=27	67%. OR=3 (0.9,10.2); p=0.1; n=27
Other	35%. OR=0.7 (0.2,2); p=0.4; n=37	58%. OR=2.1 (0.7,6.5); p=0.2; n=36
**Competition environment**	***Ref: Indoor****; 21%. n=43*	***Ref: Indoor****; 26%. n=42*
Outdoor	61%. OR=6 (2.4,14.9); p<0.001; n=62	80%. OR=11.3 (4.3,29.3); p<0.001; n=60
**Competition category, outdoor**	***Ref: Power****; 75%. n=20*	***Ref: Power****; 79%. n=19*
Endurance	50%. OR=0.3 (0.1,1.2); p=0.1; n=28	78%. OR=0.9 (0.2,4); p=0.9; n=27
Mixed	100%. OR=$1.4 \cdot 10^7$ (0,Inf); p>0.9; n=7	86%. OR=1.6 (0.1,18.3); p=0.7; n=7
Skill	29%. OR=0.1 (0,1); p=0.04; n=7	86%. OR=1.6 (0.1,18.3); p=0.7; n=7

Table 4 Explanatory models for cooling use in the past and in Tokyo, with participant characteristics as predictor variables. Percentage of "Yes"-responders, OR (95% CI), p-value and *n* respondents per group are presented. Abbreviations: OR, odds ratio; CI, confidence interval; Ref, reference group.

	**Cooling use in the past**	**Cooling use in Tokyo**
**Sex**	***Ref: Female****; 64%. N=53*	***Ref: Female****; 82%. n=45*
Male	68%. OR=1.2 (0.5,2.7); p=0.7; n=50	72%. OR=0.6 (0.2,1.6); p=0.3; n=43
**Age**	OR=1 (1, 1); p>0.9; n=103	OR=1 (1, 1.1); p=0.1; n=88
**Continent**	***Ref: Europe****; 62%. n=39*	***Ref: Europe****; 82%. n=28*
Africa	50%. OR=0.6 (0,11.4); p=0.7; n=2	100%. OR=$2.5 \cdot 10^7$ (0, Inf); p>0.9; n=2
Asia	72%. OR=1.6 (0.6,4.3); p=0.3; n=40	72%. OR=0.6 (0.2,1.9); p=0.3; n=39
North-America	50%. OR=0.6 (0.1,3.6); p=0.6; n=6	50%. OR=0.2 (0,1.5); p=0.1; n=6
Oceania	100%. OR=$2.7 \cdot 10^7$ (0,Inf); p>0.9; n=10	100%. OR=$2.5 \cdot 10^7$ (0, Inf); p>0.9; n=10
South-America	17%. OR=0.1 (0,1.2); p=0.1; n=6	67%. OR=0.4 (0,6.1); p=0.5; n=3
**Impairment category**	***Ref: VI/II/Pilot****; 59%. n=22*	***Ref: VI/II/Pilot****; 61%. n=18*
Spinal cord injury	72%. OR=1.8 (0.5,7); p=0.4; n=18	81%. OR=2.8 (0.6,13.7); p=0.2; n=16
Limb deficiency	68%. OR=1.5 (0.4,5); p=0.5; n=25	71%. OR=1.5 (0.4,5.8); p=0.5; n=24
Other	65%. OR=1.3 (0.4,3.9); p=0.7; n=37	90%. OR=5.5 (1.2,26.1); p=0.03; n=29
**Competition environment**	***Ref: Indoor****; 46%. n=41*	***Ref: Indoor****; 58%. n=31*
Outdoor	79%. OR=4.4 (1.8,10.6); p<0.001; n=62	88%. OR=5.2 (1.7,15.3); p=0.003; n=57
**Competition category, outdoor**	***Ref: Power****; 80%. n=20*	***Ref: Power****; 82%. n=17*
Endurance	79%. OR=0.9 (0.2,3.9); p=0.9; n=28	89%. OR=1.7 (0.3,10); p=0.5; n=27
Mixed	100%. OR=$1.1 \cdot 10^7$ (0, Inf); p>0.9; n=7	100%. OR=$2.5 \cdot 10^7$ (0, Inf); p>0.9; n=7
Skill	57%. OR=0.3 (0.1,2.2); p=0.2; n=7	83%. OR=1.1 (0.1,13.5); p>0.9; n=6

Table 5 Explanatory models strategy use in Tokyo, with EHI symptom history as predictor variables. Percentage of "Yes"-responders, OR (95% CI), p-value and *n* respondents per group are presented. Abbreviations: OR, odds ratio; CI, confidence interval; Ref, reference group.

	**Heat acclimation use in Tokyo**	**Cooling use in Tokyo**
**Heat-related illness symptoms in the past**	***Ref: No****; 44%. n=36*	***Ref: No****; 60%. n=30*
Yes	66%. OR=2.4 (1,5.9); p=0.045; n=53	86%. OR=4 (1.3,12.1); p=0.01; n=49

Table 6 Explanatory models for heat-related symptom occurrence in Tokyo, with strategy use in Tokyo as predictor variables. Percentage of "Yes"-responders, OR (95% CI), p-value and *n* respondents per group are presented. Abbreviations: OR, odds ratio; CI, confidence interval; Ref, reference group.

	**Heat-related illness symptoms in Tokyo**
**Heat acclimation use in Tokyo**	***Ref: No****; 14%. n=21*
Yes	25%. OR=2 (0.5,8.9); p=0.4; n=32
**Cooling use in Tokyo**	***Ref: No****; 0%. n=11*
Yes	19%. OR=$2.7 \cdot 10^7$ (0, Inf); p>0.9; n=36
